# Impact of equine interactions on human acute pain perception: Two cross sectional studies

**DOI:** 10.1177/20494637241302391

**Published:** 2024-11-29

**Authors:** Gwyneth Doherty-Sneddon, Roberta Caiazza, Emilia Pawlowska, Quoc Vuong

**Affiliations:** 1 School of Psychology, Dame Margaret Barbour Building, 5994 Newcastle University, Newcastle upon Tyne, UK; 2 Cavallo Therapy, Psychological Consultancy RC LTD, Seaton Sluice, Whitley Bay, UK; 3 Biosciences Institute, 5994 Newcastle University, Newcastle upon Tyne, UK

**Keywords:** Acute pain, Animal-Assisted Therapy, Equine Assisted Pain Reduction, pain perception, pain management

## Abstract

**Background:**

Research has demonstrated the effectiveness of Animal-Assisted Therapy, usually involving dogs, as a way to reduce pain in inpatient and outpatient populations. Here two studies investigate the effectiveness of interacting with horses for reducing human acute pain perception.

**Methods:**

In Study 1, a blood-pressure cuff was used to administer acute ischaemic pain to 70 adult participants, who were allocated to one of three groups: Equine Assisted Psychotherapy (EAP), Horse Interaction without EAP (HI), and a Control (no horses present). All participants engaged in an activity (finding a horse treat) in a large, enclosed arena. The dependent variable was the subjective pain rating (scale 0-10) of the participant in response to moderate pain induced pre- and post-activity. In Study 2, 53 adult participants were recruited and allocated to either an Equine Assisted Learning (EAL) Group or a Control Group. The same paradigm was used. Following the activity sessions, qualitative data was elicited from the participants regarding their insights and feelings. It was hypothesized that any interaction with horses would significantly reduce an individual’s perception of pain.

**Results:**

In both studies, planned paired-samples t-tests showed significant reductions in pain ratings from pre-activity to post-activity in the EAL, EAP and HI groups (large and medium effect sizes) but not the Control groups. Thematic analysis of the qualitative responses showed an overwhelmingly positive array of responses from those who interacted with the horses, for example, feeling relaxed and happy during the activity.

**Conclusion:**

Interactions with horses can reduce acute pain perception. Distraction, physiological changes, and positive emotions are discussed as possible underlying mechanisms. It remains to be seen how this could be more widely applied, for example, in relation to chronic pain.

## Introduction

Chronic pain, defined as pain lasting over 3 months, is a global public health problem that impairs well-being and quality of life.^1^ Treating chronic pain can involve pharmacology; however, such treatments can cause side effects like hyperalgesia and addiction.^2^ Recent research promotes alternative therapies in conjunction with biomedical treatment,^3^ which can reduce pain more effectively than medication alone.^4^ The current study is the first of its kind to investigate whether interacting with horses can reduce pain perception. This horse-interaction involves ground-based activities with horses and does not involve riding. Given the novelty of the study, we investigated the effect of horse-interaction on acute pain perception rather than testing a patient group with chronic pain. Our longer-term research aims are to investigate interactions with horses in the treatment of chronic pain, in which the necessity of non-pharmaceutical interventions becomes of particular importance.

Recognition of the importance of psychosocial factors in pain perception has led to development of psychotherapies for chronic pain.^5^ One form of psychosocial intervention for pain management is Animal-Assisted Therapy (AAT). AAT can improve various health conditions, often more effectively than traditional treatments.^6,7^ Interaction with therapy animals can trigger the release of β-endorphins^8,9^ – neuropeptides that act as natural painkillers.^10^ The Dynamic Model of Affect (DMA^11^) is also consistent with this explanation, hypothesizing that the ability to sustain positive states decreases vulnerability to pain. Studies supporting this model demonstrate that AAT is linked to decreased stress, mood disturbance and fatigue, alongside reduction in pain perception (PP).^8,12^

Horses have been integrated into therapeutic interventions (including therapeutic riding and hippotherapy) to treat various conditions including chronic pain.^13,14^ In a review of the literature, White-Lewis et al. (2017)^15^ report that while 94% of the studies they looked at reported significant benefits to people with chronic pain, the majority of studies lacked methodological rigour (e.g. not including an adequate control) and therefore results must be treated with some caution.

Equine Assisted Learning (EAL), a non-riding (ground-based) activity involving horses, has not yet been used in the treatment or study of pain. Equine Assisted Psychotherapy (EAP) includes a psychotherapeutic component.^16,17^ As a social prey animal, horses are attentive to environment changes, including micro and macro body movements in humans, as seen in the classic study of Clever Hans.^18^ Some researchers have suggested that horses are instinctively attentive to mood,^19^ reacting sensitively to humans.^20^ This can have psychological benefits for humans, including improved communication, confidence and emotion regulation.^21^

The benefits of interacting with horses may subsequently affect cognitive and psychosocial factors that moderate acute pain perception. It is proposed that task and pain processing compete for attentional resources,^22,23^ thus pain-related brain activation can be reduced through interactive (rather than passive) distraction.^24^ Psychosocial factors can bias attention away from pain sensation, aiding pain management^25–29^ in both chronic and acute pain.

The two studies reported here investigate whether interacting with horses with psychotherapeutic input (EAP) and without psychotherapeutic input (general horse-interaction and EAL) can reduce acute pain perception. Pain was administered to participants using a blood-pressure cuff^30^ and pre- and post-activity pain ratings were taken. Follow-up questionnaires and interviews explored participants’ subjective experiences. We hypothesized that EAL, EAP and HI activities would lead to significant reductions in acute pain ratings.

## Study 1

### Method

#### Participants

Participants were recruited via volunteer sampling, using word-of-mouth and social media. Two rounds of data collection for Study 1 were carried out in autumn 2022 and spring 2022. Seventy participants (26 males, 44 females) took part. Their mean age was 36.0 years old (SD = 16.5 years), with a range of 18 – 70 years. Inclusion criteria were that participants were over 18 and had no known vascular disease (because of the method of pain induction). Participants were randomly allocated to one of three experimental groups, with the constraint that a similar age range were present in each group to control for age differences. Numbers of males and females were roughly equivalent across each of the three groups. Existence of a chronic pain condition was not an exclusion criterion. Because of the sampling methods used, some participants had some experience with horses and others had none. The descriptive statistics of the participants for each group is presented in Table 1.

**Table 1.** Descriptive statistics of participants in each experimental group.

	EAP	Control	Horse-interaction	Overall
Mean age (years)	35.0	33.0	39.0	36.0
SD age (years)	17.4	14.8	17.3	16.5
Male	8	9	9	26
Female	16	13	15	44
Total participants	24	22	24	70

This study was approved by Newcastle University’s Faculty of Medical Sciences ethics committee (reference code: 2257/17268), which conformed to the 1964 Declaration of Helsinki on Human Experimentation. Participants were fully informed about the pain induction and (potential) interaction with horses, and provided signed consent form. They were also required to sign an additional contract and medical history form that was mandatory for insurance purposes for the equine sessions.

##### Therapy horses

Two therapy horses took part in the EAP and the HI sessions. There was one Irish Draft mare (16.2 hands – 1.68 m height to withers/shoulder; 9 years old) and one Cross Breed mare (15.1 hands – 1.55 m height at withers/shoulder; 14 years old). The involvement of these horses in the research was overseen by the Cavallo Therapy Health and Safety Policy Statement, ensuring compliance with the regulations outlined in the UK Animal Welfare Act of 2006 and the ethical guidelines stipulated by the Equine Assisted Growth and Learning Association (EAGALA) (an international organization offering equine-assisted therapy training). The welfare of the horses was the responsibility of Cavello Therapy personnel who also owned them privately. The equine specialist and EAP practitioner have close bonds with both horses. In addition, both horses are used at least weekly for clinical EAP sessions and are accustomed to meeting strangers and interacting with them.

##### Researchers and facilitators

Sessions for all groups were led by a qualified EAP practitioner/clinical psychologist (author RC), who was present throughout each session for all participants. In addition, an equine specialist was present during the sessions involving horses and a group of seven research assistants was responsible for collecting data from all participants, including the pain measurements. Only one research assistant was present in any given session.

#### Design

This study was a mixed methods design, using both a quantitative and qualitative approach. The experimental component had a 2 time points × 3 groups mixed design. The two time points were tested within-subjects (pre-activity vs post-activity). The first group was the Equine Assisted Psychotherapy Group (EAP), in which participants carried out activities with the two horses under psychological guidance. The second group was the Horse Interaction (HI) Group, in which participants carried out activities with the two horses without psychological guidance. Finally, participants in the Control Group carried out activities in the horse arena without the horses. For each time point, we measured participants’ pain rating to moderate pain.

Data collection occurred in the spring of 2022 (Round 1) and the autumn of 2022 (Round 2). We received interesting verbal ad hoc feedback from participants during Round 1 and decided to try to capture this more systematically during Round 2. All participants from Round 2 were invited to respond to a follow-up questionnaire to elicit their experiential reflections on the session. An epistemological approach, critical realism^31^ was taken to allow each participant’s response to be looked at independently, focussing on the individual and being mindful of any differences between responses. Themes were identified using a ‘cutting and sorting’ technique, whereby different quotes were gathered and arranged into groups that fit together,^32^ leading to the emergence of four themes.

#### Materials

##### Pain induction

Pain was safely induced using a Primacare DS-9181-BK Professional Aneroid Sphygmomanometer (blood-pressure cuff). The sphygmomanometer induces ischaemic pain by applying pressure on the arm where the cuff is placed. This is a method for induction of acute pain that has been used experimentally to study pain perception in rowers.^30^

##### Questionnaires

Before starting the study, each participant completed a demographic questionnaire. An online follow-up questionnaire consisted of four questions addressing how the participants felt, what they could remember about their experience, as well as any moments that stood out and any notable differences they observed from pre-session to post-session. The questions asked were designed to target the aspects of the experience that were relevant to the hypothesis while also allowing participants to recall as much as they could/wanted to. It was only sent to those that participated in the second round of data collection (*n* = 25).

##### Arena for activities

An indoor equestrian exercise arena was used for all sessions. It was approximately 20 m × 20 m. It has a roof but is not heated and therefore inside is roughly equivalent to external temperatures, although protected from the wind and rain. No record was kept of daily weather conditions, but participants were asked to dress according to weather conditions, for example, extra layers if it was cold so that they were comfortable. There were a few objects in the arena area – stacked jumping poles in the viewing gallery which were visible from the arena, a mounting block, and two cones. The cones and mounting block could be explored by the participants if they chose to do so.

##### Baseline meansure of pain perception

We first measured the pressure (in mm Hg) leading to pain threshold and pain tolerance. These baseline measurements were taken before the start of the session outside of the arena (with no horses present), and used to calculate the pressure to induce moderate pain. The cuff was placed above the elbow on the participant’s non-dominant arm.^30^ Pressure was slowly applied at the cuff by gently pumping the sphygmomanometer to induce ischaemic pain. To obtain the pain threshold of the participant, the researcher gradually pumped the cuff and instructed the participant to indicate when the sensation became ‘uncomfortable’, that is, 1 out of 10 on a 10-point numeric rating scale. To obtain pain tolerance, the researcher continued to slowly increase pressure at the cuff until the participant indicated that the sensation was ‘unbearable’, that is, 10 out of 10. The cuff was then deflated immediately after to release the pressure on the arm. This process was repeated, and the average pressure leading to pain threshold and pain tolerance was calculated for each participant.

The pressure required to induce moderate pain for each participant was then calculated using the following equation: (average pain tolerance – average pain threshold) × 0.6 + average pain threshold. This equation allowed us to normalize each individual pain range between threshold and tolerance, and taking 60% of that range (6 on a 10-point scale). Most studies report a numeric rating between 4 and 8 corresponded with moderate pain (e.g. Vuong et al., 2018). The calculated moderate-pain pressure for each group were as follows: EAP (*M* = 196.7 mmHg, *SE* = 10.2 mmHg), HI (*M* = 215.1 mmHg, *SE* = 10.3 mmHg) and Control (*M* = 199.3 mmHg, *SE* = 9.6 mmHg).

#### Procedure

Participants were tested in cohorts of one to five individuals on a given testing session. The timeline for each cohort was as follows:

• Pre-activity measures taken by research assistants (in horsebox)
• Cohort safety instruction and familiarization (in arena) with EAP practitioner in all groups.
• Cohort activity (in arena: all participants in group, with the 2 horses if in EAP or HI) with EAP practitioner in all groups.
• Individual activity (in arena: 1 participant at a time with the 2 horses) with EAP practitioner in all groups. Other cohort members leave arena and wait outside arena building with a research assistant.
• Each participant has a blood-pressure cuff applied by research assistant before returning to the arena for their individual session (the participant to go first in the individual session has cuff applied before entering arena with the cohort)
• Individual post-activity measures (in arena: 1 participant at a time, with the 2 horses if in EAP or HI) and with EAP practitioner in all groups.

Impact of cohort size was beyond the scope of the current study, but varied in a similar way across the three experimental groups. All participants in a session were assigned to the same experimental group. We first measured the pre-activity pain rating to moderate-pain pressure individually for each participant in the group outside of the test arena. Pre-activity ratings were taken in a horsebox near the arena. Other members of each participant’s cohort were present but were free to leave the box and walk about outside if they wished.

The EAP practitioner and one of the researchers then took all participants for that session into the viewing gallery of the arena. Participants had 5 min to familiarize themselves with the arena and the two horses (if present) from the gallery. During this time, the participants received a safety briefing regarding their interactions with the horses (if in EAP or HI groups) and were then invited to enter the main part of the arena. The horses were at liberty in the arena. Halters were close by for safety reasons but the horses were not wearing them during their interactions with participants.

During the activity, all instructions were given verbally by the EAP practitioner and the participants were observed by the psychologist and an equine specialist who were present in the arena at all times. The research assistant responsible for taking pain ratings remained in the viewing gallery for the majority of the session, only entering to take the individual ratings as individuals completed their session. Instructions were given to the participants for the task they were asked to do. Participants were told they would have up to 10 min to do the task as a group and then a further 10 min to try the task as an individual while the rest of the group stepped outside (normally back to the horsebox or they were free to walk around outside and near to the arena building. Participants were introduced to a horse treat, subsequently concealed within the arena’s surface. In order to complete the activity, they were guided to visualize the arena as a sprawling field where they had unintentionally lost their keys (symbolized by the horse treat). Their objective revolved around successfully recovering these ‘keys’/treats. This task is one that the author RC had experienced while training as an Equine Assisted Psychotherapist with EAGALA (an international organization offering equine-assisted therapy training). The point of the task was to operationalize a cooperation between the horses and the participants. The treats were approximately 2 cm long and brown in colour. The substrate of the arena was also brown and therefore finding the treat was challenging. The session leader hid the treat for each individual and attempted to make the task of comparable difficulty across participants. If the treat was found the participant was allowed to feed this to the horse they were interacting with at the time. This feeding was considered part of the possible interactions that could happen during the session between participant and horse.

The activity performed by the participants in each Group was as follows: In the EAP Group, the participants helped horses find treats hidden in the arena. Participants attempted to get the horses to follow them at liberty while looking for the treat. There were no rules apart from everything was done safely respecting the horses’ space and willingness to engage. Participants could interact with one or both horses. Interactions were very fluid, sometimes both horses engaged with the participant at a time, sometimes one horse would observe or wander off while the other interacted with the participant. Simultaneously, the clinical psychologist posed reflective questions and led a concise EAP session. This session aimed to delve into participants’ current thoughts and emotions associated to the search task and their interaction with the horse, in order to support the participant’s ability to reflect on the impact of the experience and develop better self-awareness in the here-and-now. In the HI Group, the participant assisted the horses in finding the treat hidden in the arena. However, no direction was given by the psychologist, and EAP did not take place. In the Control Group, the participant was tasked with finding the hidden treat with no horses present. The EAP practitioner was present throughout and provided some verbal encouragement and interaction in both group and individual sessions. In addition, participants interacted with one another during the group part of each session in all groups. After 10 min of the group attempting the task each participant then also had a further 10 min to complete the task without the rest of the group. One participant completed the task before this duration (a control group participant), but most took the full 10 min and/or did not complete the task.

Once participants completed their activity, we measured their post-activity pain rating to moderate-pain pressure while they were still in the arena. The horses remained in the arena in the EAP or HI groups during the post-activity pain rating. The equine specialist and the EAP practitioner were also still present, but unlike the pre-activity ratings no other participant group members were present. This was the same across all conditions. As before, the researcher fitted the blood-pressure cuff to the participant’s non-dominant arm and gradually inflated it to the moderate-pain pressure. The participant was asked to verbally rate the extent to which the cuff felt painful on the same scale. This rating was repeated twice, and an average calculated.

The follow-up questionnaire was sent by email to each participant (in round 2) the day after they took part in the study. The researcher taking pain ratings during the sessions alternated between testing days but remained consistent across all three Groups for each day to allow the horses to be familiar with those in the arena. The researcher assistants had all met both horses on 1 occasion prior to the research beginning, during which they interacted with them at liberty. During the testing sessions they did not interact with the horses but were present only to take readings from the participants. The Experimental Groups were ordered and partially counterbalanced, starting and ending with one of the horse conditions and the Control Group always in the middle, to allow for the horses to rest between the EAP and HI sessions.

#### Data analysis

The average pain ratings before and after the activity were submitted to a 2 (Time Point: pre-activity, post-activity) × 3 (Group: EAP, HI Control) mixed analysis of variance (ANOVA), with Time Point as the within-subjects variable and Group as the between-subjects variable. Given our a priori hypotheses, we used planned paired *t*-tests to determine if there was a significant decrease in pain rating after the activity for each experimental Group. A significance level of *p* = .05 was adopted for all statistical analyses reported, and all analyses used a one-tailed test.

The responses to the follow-up questionnaire were coded, and thematic analysis was used to extract four themes. Of the 25 participants who were included in the second round of data, only 14 completed the questionnaire (HI Group: *n* = 9; EAP Group: *n* = 4; Control Group: *n* = 1). Given the low numbers of responses, the thematic analysis is exploratory and is limited to considering in any depth the groups who interacted with horses rather than the control group experience. Participant responses were numbered 1-14, with P1-P9 (HI), P10-P13 (EAP), and P14 (Control). The epistemological approach, critical realism,^31^ was taken to allow each participant’s response to be looked at independently, focussing on the individual and being mindful of any differences between responses. Themes were identified using a ‘cutting and sorting’ technique, whereby different quotes were gathered and arranged into groups that fit together,^32^ leading to the emergence of four themes.

### Results

The descriptive statistics for pre-activity and post-activity pain ratings for each Group are shown in Table 2.

**Table 2.** Mean and standard deviation of pain rating across participants before and after activity for each group.

	EAP		HI		Control	
	Mean	SD	Mean	SD	Mean	SD
Pre-activity	6.35	1.53	6.19	1.83	5.89	1.94
Post-activity	4.81	2.07	5.06	2.14	5.52	1.76

The 2 × 3 ANOVA showed that there was a significant main effect of Time Point, *F*(1, 67) = 24.67, *p* < .001, $\eta_{p}^{2}$ = 0.27. The average post-activity pain rating (*M* = 5.13, SE = 0.24) was significantly lower than the average pre-activity pain rating (*M* = 6.14, SE = 0.21). There was no main effect of Group (*p* = .97). The interaction between Time Point and Group approached significance, *F*(2, 67) = 2.81, *p* = .07, $\eta_{p}^{2}$ = 0.08. Given the a priori prediction that subjective pain perception would decrease after activities involving the horses but not after activities without the horses, planned *t*-tests were used to explore the marginal interaction. Pain rating was significantly lower in the post-activity compared to pre-activity measurement for the EAP Group, *t*(23) = 4.76, *p* < .001 (one-tailed test). The effect size for this group (*d* = 0.97, 95% CI [0.48 – 1.45]) was found to exceed Cohen’s (1988) convention for a large effect (*d* = 0.80). The same pattern was found for the HI Group, *t*(23) = 3.06, *p* < .01 (one-tailed test), with a medium effect size (*d* = 0.62, 95% CI [0.18 – 1.06]). There was no significant difference between the pre-activity and post-activity pain rating for the Control Group, *t*(21) = 1.00, *p* = 0.16.

#### Qualitative analysis

**Figure 1.**
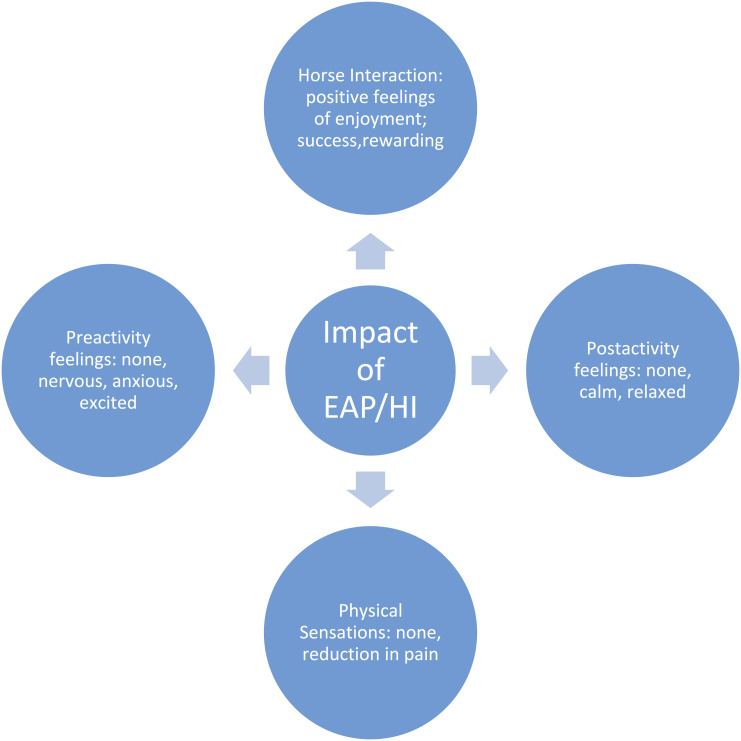
Thematic map of study 1 participant self-reported experiences of EAP and HI.

Only 14 of the participants responded to the post-session questionnaire regarding their experience of their sessions. While this is only around 23% of the participants and almost all were from the EAP and HI groups, we considered that their responses provided valuable insights into how participants interacting with horses experienced the activity. An analysis of their responses is given in this section. Four themes were identified relating to EAP/horse-interaction, shown in the thematic map (Figure 1). Each theme raised important points regarding the individual experience of participation in the study. ‘Physical sensations’ describe any physical observations that participants noted throughout the experiment. ‘Pre-activity feelings’ describe the emotions experienced by participants prior to taking part. ‘Post-activity feelings’ describe the emotions experienced by participants after taking part. Finally, ‘horse interaction’ describes any observations, emotions, and feelings that those in the EAP and HI Groups noted during their session. The most relevant examples from the responses have been used to embody each theme.

##### Physical sensations

Throughout the questionnaire, participants were prompted to recall various parts of their experience. Four participants (HI Group: *n* = 3, Control Group: *n* = 1) noted observations of physical sensations, with those who were in the HI Group highlighting a conscious observation of pain reduction: ‘*The pain I felt…significantly less at the same pressure as the pre-task levels*’ – P4, ‘*my pain threshold in the arena was so much higher*’ – P3. The response from the individual in the Control Group noted feeling ‘*physically nothing*’ – P14, during their experience.

##### Pre-activity feelings

When prompted to recall what they felt during the experience, participants tended to respond by separating their responses into pre-activity and post-activity feelings. Referencing pre-activity feelings (EAP: *n* = 1, Control: *n* = 1, HI: *n* = 9), responses from those in the EAP and HI Groups indicated nervousness/anxiety as a focal point of their pre-activity experience: ‘*Before I was nervous, a bit anxious not knowing what to expect*’ – P9. This nervousness was sometimes reported as coming from an apprehension of interacting with the horses: ‘*I felt a bit scared about being around such large and strong animals*’ – P11. In addition to this, two participants in the HI Group noted their positive feelings of excitement and curiosity: ‘*I felt very excited before the interactions with the horses*’ – P5, ‘*Initially very curious about the whole experiment*’ – P3. The Control Group participant responded ‘*None*’ – P14, to the questions inquiring about pre-activity feelings.

##### Post-activity feelings

Of those who mentioned post-activity feelings (EAP: *n* = 2, HI: *n* = 7), all responses were positive, with a specific recall of being relaxed, calm, and happy after interaction with the horses: ‘*Felt more relaxed and confident*’ – P7, ‘*I came away feeling really happy*’ – P8, ‘*calmer and head clearer*’ – P13, and ‘*This made me quite emotional and I thought about this for the rest of the 18 days. The notion that my company is enjoyable and worth having without any other motive is something I haven’t really believed before so it took me by surprise*’ – P10. Like pre-activity feelings, the participant in the Control Group also responded ‘*None*’ – P14, when asked about post-activity feelings.

##### Horse-interaction

Although there was not a question tailored directly to how participants felt around the horses, many responses (EAP: *n* = 3, HI: *n* = 8) highlighted positive experiences and effects of the horse presence in the EAP and HI Groups: ‘*I really started to relax once I was with the horses and really enjoyed interacting with them*’ – P8. With reference to the activity itself, responses indicated the emotions associated with success within the task: ‘*It was amazing to feel them (horses) respond and follow me*’ – P3, and ‘*feel really good when Gracie followed me*’ – P2. A rewarding feeling was also associated with task success: ‘*I felt rewarded when the horses responded to me*’ – P11.

## Study 2

### Method

#### Participants

Participants were recruited via volunteer sampling, using a research participation scheme and word-of-mouth. All testing was done in the autumn/winter of 2023-24. To sign up participants were required to be over the age of 18 and have no history of vascular disease. None of these participants had taken part in Study 1. Fifty-three participants (43 females, 9 males, 1 non-binary) with a mean age of 20.08 years old (SD = 4.46 years) took part. Prior to arriving at the site of the study, 26 participants were allocated to the Control Group, and 27 were allocated to the EAL Group. Age and gender were controlled for by ensuring a similar distribution in each group. This study was approved by Newcastle University’s Faculty of Medical Sciences ethics committee (reference code: 2624/35236) and participants were required to provide informed consent regarding the pain induction and potential interaction with horses. They also completed a medical history form that was required for emergency purposes for the equine-assisted sessions.

##### Equine assisted learning ponies

Ten riding school ponies took part in the EAL sessions at a Riding Centre near Newcastle. They were all in work with riding lessons and were fit and well. Their sizes ranged from 12.0 hands to 15.1 hands. The youngest pony to take part was 9 years old and the oldest was 25 years old. While there was a mix of mares and geldings, they all took part in the EAL sessions in same sex friendship pairs. The study received Animal Welfare and Ethical Body approval Project ID: 1063. An inclusion/exclusion checklist (appendix 1) was applied each time a pony took part and any welfare/health issues discussed with the Riding Centre if they arose.

##### Researchers and facilitators

Sessions for all groups were led by a qualified EAL practitioner/Development Psychologist (author GDS) who was present throughout each session for all participants. In addition, a group of three research assistants were responsible for collecting data from all participants, including the pain measurements.

#### Design

This study was a mixed methods design, using both a quantitative and qualitative approach. The experimental component used a 2 Time Points × 2 Groups mixed design. The two Time points were tested within-subject (pre-activity vs post-activity pain rating). The two Groups were between-subject (EAL Group vs Control Group), so participants were randomly allocated to one of two groups. The first group was the EAL Group, in which participants carried out activities with guidance from the EAL facilitator, in the presence of two ponies. The Control Group carried out activities in the horse arena without the ponies present. The EAL practitioner was present throughout all sessions for both groups. Some verbal interaction occurred in both groups between her and the participants during their group and individual activity. In addition participants interacted with one another during the group part of each session. For each time point, we measured the participants’ pain rating to moderate-pain induction. The qualitative portion of the study consisted of short individual interviews with each participant straight after their individual session. An epistemological approach, critical realism,^31^ was taken so that each participant’s response was looked at independently, focussing on the individual as well as any differences between responses within as well as between groups. Thematic analysis based on Braun and Clarke’s six stage framework (2006)^33^ was used to analyze and code each transcript.

#### Materials and procedure

Materials, measures and procedure were all the same as Study 1 with the following exceptions. The calculated moderate-pain pressure for each group were as follows: EAL (*M* = 156.9 mmHg, SE = 7.3 mmHg), and Control (*M* = 167.7 mmHg, SE = 7.4 mmHg). The activities were carried out in a larger indoor arena (40 m × 22 m). It again had a viewing gallery and there were a few objects within the arena as in study 1 which participants sometimes used in their interactions with the ponies. The facilitator of the activity sessions was the EAL practitioner. The post-activity questionnaires were replaced by semi-structured interviews conducted immediately after the activity sessions. These consisted of four open questions: How did you feel during the session? What can you remember about the experience? Were there any moments that stood out? Did you observe any notable differences from pre-session to post-session?

#### Data analysis

For each participant the average pain ratings for before (pre-activity) and after (post-activity) the test sessions were calculated. A 2 (Group: EAL, Control) × 2 (Time Point: pre-activity, post-activity) mixed analysis of variance (ANOVA) was conducted, with Group as the between-subjects variable, and Time Point as the within-subject variable. Given our hypothesis, we used planned paired *t*-tests to determine if there was a significant decrease in pain rating post-activity for the EAL and Control Groups and adopted a significance level of *p* = .05 for the statistical analyses reported, using a one-tailed approach. The interview transcripts for each participant were coded, and thematic analysis based on Braun and Clarke’s six stage framework (2006)^33^ led to the emergence of four themes. Of the 53 participants whose quantitative data was collected, 45 were successfully interviewed (EAL Group, *n* = 24, Control Group, *n* = 21). The eight without interview data were due to insufficient time for them to be interviewed or because recording equipment failed. The participant’s interview number corresponded with their participant ID for the quantitative data collection and a letter was added depending on whether they participated in an EAL session (e.g. P1H) or were part of the Control Group (e.g. P6C).

### Results

#### Quantitative analyses

The descriptive statistics for pre-activity and post-activity pain ratings are shown in Table 3.

**Table 3.** Mean and standard deviations of pain ratings before and after session by experimental Group.

	EAL		Control	
	Mean	SD	Mean	SD
Pre-activity pain rating	6.45	1.62	6.28	1.93
Post-activity pain rating	5.05	1.35	6.54	1.83

The 2 × 2 ANOVA showed that there was a statistically significant main effects of Time Point, *F*(1, 51) = 20.04, *p* < .01. The average post-activity pain rating was significantly lower than the average pre-activity pain ratings, with a large effect ( $\eta_{p}^{2}$ = 0.28). There was no main effect of Group. The interaction between Time Point and Group was statistically significant, *F*(1, 51) = 41.79, *p* < .01, with a large effect ( $\eta_{p}^{2}$ = 0.45). Given the prior assumption that the pain rating would decrease across Time Point in the EAL Group but not in the Control Group, planned *t*-tests were used to explore the interaction. Pain rating was significantly lower in the post-activity compared to pre-activity measurement for the EAL Group, *t*(26) = 8.18, *p* < .01, and the effect size for this group (*d* = 0.89 95% CI [1.00 – 2.14] was found to exceed Cohen’s (1988)^34^ convention for a large effect (*d* = 0.80). There was no significant difference between the pre-activity and post-activity pain rating for the Control Group, *t*(25) = −1.33, *p* = 0.20 (two-tailed test).

#### Qualitative analysis

**Figure 2.**
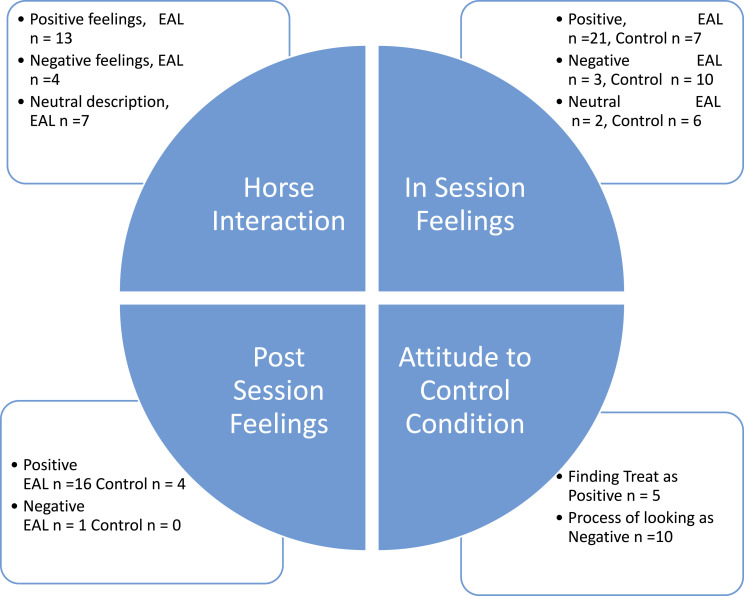
Thematic map of study 2 participant self-reported experiences of EAL and control.

Forty-five (Control Group: *n* = 21, EAL Group: *n* = 24) of the participants were interviewed post-session regarding their experience and an analysis of their responses is given in this section. Four themes were identified, two of which related to both the EAL and Control Groups, shown in the thematic map (Figure 2).

Each theme allowed us to dissect the experiences of those participating in our study allowing us to examine differences between individuals within and across conditions. ‘In-Session Feelings’ describes any feelings that the participants reported feeling during the group and individual sessions. ‘Horse Interaction’ describes any experiences related to the horses discussed by the EAL Group, such as their thoughts on their own interactions with the horses or how they felt observing the ways in which the horses interacted with one another and the environment. The theme ‘Attitude Toward Control Task’ applies to the Control Group’s insights into how their task impacted them, even though there were no horses present. ‘Post-Session Effects’ refers to any changes reported by the participants as a result of carrying out the activity. The most relevant quotes from the transcripts have been used to represent each theme.

##### In-session feelings

To begin the interview, the participants were asked how they felt during the experience. These could be divided into positive, neutral or negative experiences from a participant perspective. A total of 28 participants (EAL Group: *n* = 21, Control group: *n* = 7) described their experience as positive, including reports of feeling relaxed and happy, as well as some describing their experience as fun, enjoyable, exciting or interesting. Some participants expressed several different emotions and the incidence of each is discussed below. The experience of feeling calm, relaxed or chilled was reported by 18 participants. For example, P2H said: ‘[The experience made me feel] …quite relaxed and happy’. Moreover, P50H said: ‘The horses were really calm and made me feel calm’. And P34H said the experience was ‘quite calming and that [the ponies] were nice to be around’.

There were also instances of these feelings reported by the Control Group such as P26C who felt ‘quite calm’ and P44C who felt ‘pretty normal and chill’. Twelve participants (EAL Group: *n* = 9, Control Group: *n* = 3) reported feeling happy or felt like they had a good or nice experience. Those in the EAL group often attributed their feelings of happiness to their interaction with the horses: ‘…the horses made me happy’ – P2H, ‘It was very nice to do’ – P18H. Those in the Control Group often noted feeling happy due to completing the activity objective and finding the treat: ‘I found [the treat] very quickly so I was very happy’ – P6C.

There were also instances of EAL participants describing their experience as fun (*n* = 3), enjoyable (*n* = 3), exciting (*n* = 2), and interesting (*n* = 1). There were 8 participants who didn’t express feeling a strong emotional response within the session and described feeling fine or indifferent during the session (EAL Group: *n* = 2, Control Group: *n* = 6). Participants P7C and P10C also described feeling neutral about the experience but later noted feeling frustration, suggesting that some participants had a more nuanced grasp of their feelings and were able to separate their emotional state from negative cognitive feelings they were experiencing as a result of the task.

A total of 13 participants (EAL Group: *n* = 3, Control Group: *n* = 10) experienced negative feelings during the session. One participant (P16H) described the experience as ‘a bit scary’ but also ‘kind of fun’ overlapping between having positive and negative in-session feelings. Including participant P16H, 3 participants noted feeling stressed or scared during the session (EAL Group: *n* = 2, Control group: *n* = 1). For example, participant P29C said they felt ‘a bit stressed because [they] couldn’t find the treat’. Two Control Group participants recalled feeling awkward or embarrassed during the session, while another 2 Control Group participants reported feeling bored. Six Control Group participants expressed negative cognitive feelings which included confusion (*n* = 4) and frustration (*n* = 2).

##### Horse-interaction

Although the interview questions did not specifically ask the participants about their interactions with the horses, when asked what they could remember or if there were any moments which stood out to them, all those in the EAL condition (*n* = 24) described their interactions or what they observed of the horses interacting with each other. It is useful to note that the interview began by asking the participants about their feelings which may have influenced them to answer subsequent questions in a way which centred around or justified their previous answer. Thirteen participants attributed their positive feelings to the horses: ‘The horse made me happy’ – P2H; ‘Definitely more relaxed when I’m petting them’ – P13H; ‘Horses were really calm and made me feel calm’ – P50H; ‘I feel like I really bonded with them when they walked with me’ – P38H, ‘When I got the horse to follow me I felt talented’ – P18H. Four participants mentioned slightly negative experiences in regard to their interactions with the horses: ‘I was nervous but then comfortable with the horses’ – P14H; ‘Bit wary of horses’ – P17H; ‘Not a massive fan of horses, I’ve just realised’ – P12H. The rest of the EAL participants (*n* = 7) had more neutral answers simply describing what they did or how the horses acted, without going into detail about how this interaction made them feel. For example, P39H said: ‘When the horse followed me and stopped when I stopped’ and P41H talked about ‘Walking around with one of the smaller ponies around the cones’.

##### Attitudes to the control task

Unlike the EAL Group, the horses were not present for the Control Group’s session which meant that when they were asked what they could remember or if there were any moments that stood out to them, 15 participants in the Control Group discussed their feelings and attitudes towards the activity. Five participants recalled having a positive experience completing the activity because of finding the treat. For example, finding the treat was described as ‘rewarding’ – P21C and P6C said they ‘found it very quickly’ and this made them ‘very happy’. The only positive attitudes towards the task were reported by those who found the treat suggesting that it was likely the sense of accomplishment from completing the task which elicited positive feelings rather than the motions of going through the task. This is unlike the majority of the EAL Group who regarded the whole process of their interactions with the horses as a pleasant experience.

On the other hand, 10 Control Group participants who were not able to complete the task or were looking for the treat for a while (maximum 10 min), reported negative feelings towards the control task. Negative cognitive feelings previously mentioned as prominent in-session feelings experienced by the Control Group were discussed further in relation to the task specifically. One of these feelings was frustration (*n* = 2): ‘Bit frustrated walking around not being able to find it’ – P7C, ‘Slight bit of frustration as I went everywhere and didn’t find it at first’ – P10C. Two participants reported feeling bored with the task and 4 participants said they felt confused regarding the point of the task or the task itself: ‘A bit confused at what I was doing’ – P42C. For participant P29C not being able to find the treat caused them to feel a ‘bit stressed’. In addition, 2 participants expressed that having to carry out the task while being observed made them feel ‘a bit awkward’ – P22C, and ‘a bit embarrassed’ – P9C.

##### Post-session effects

When asked if they noticed any differences from pre-session to post-session, 20 participants (EAL Group: *n* = 16, Control Group: *n* = 4) reported positive emotional changes. One positive emotional effect of the session reported by 15 participants (EAL Group: *n* = 14, Control group: *n* = 1) was feeling calmer, more relaxed or less stressed. For example, P36H said: ‘I feel like a lot of my stress is gone’ and P5H said they felt ‘more relaxed and calmer’. Seven participants (EAL Group: *n* = 4, Control Group: *n* = 3) reported feeling happier or in a better mood after the session: ‘Made me feel better’ – P38H, ‘I’d say I’m probably more relaxed and in a good mood’ – P11H. Those in the Control Group who reported feeling better after the session implied that this was due to the session being over or because they felt accomplished due to completing the task: ‘Now that it’s over I feel better’ – P23C, ‘I feel a lot better because I found the treat’ – P45C, ‘I feel better about myself because I found the treat’ – P46C.

Three EAL Group participants noted that their participation in the session resulted in a physical change. Reduced pain perception was a physical change reported by two participants: ‘While they were measuring my pain response the horse was licking my hand, so I wasn’t paying as much attention to the pain’ – P5H, ‘I think the pain measurements felt less painful’ – P41H. One EAL participant reported feeling colder after the session but did not elaborate on this physical change.

Only one EAL participant expressed feeling worse as a result of participating in the session. Participant P12H reported feeling more anxious after the session due to not being comfortable interacting with the horses: ‘I was more anxious after the session. I’m not a massive fan of horses, I’ve just realised’. It is useful to note that this was the only participant in the EAL Group whose pain perception did not decrease after the session. This may suggest that an anxious state may impact the efficacy of horse-interaction in reducing pain perception.

Sixteen participants (EAL Group: *n* = 2, Control Group: *n* = 14) said they observed no difference in the way they felt before and after participating in the session.

### Discussion

The studies reported here examined the effect of horse-interaction on acute pain perception. It was hypothesized that EAP, HI (without EAP) and EAL would serve as effective means of reducing an individual’s perception of acute pain. It was predicted that those who participated in the EAP, HI and EAL groups would experience a significant reduction in pain ratings after activities involving horses, and that this reduction would not be observed in the Control groups. The results supported the hypothesis with a significant reduction in pain ratings by participants in all three horse-interaction groups but not those in the control groups. It is reasonable to suggest that being around horses (whether there is psychological guidance or not) has a significant beneficial effect on pain perception. The potential mechanisms behind this reduction may include distraction, physiological changes, and positive emotion.

The qualitative data regarding participants’ experiences of EAP, HI and EAL have similarities across the two studies. The horses and their behaviour are clearly salient to participants who readily report details of their experiences with the horses. In addition, the majority of participants report positive emotional experiences while interacting with the horses as well as feeling calmer, relaxed and happier after the sessions. It is noteworthy that participants from both studies report some nervousness before the sessions. While many then experienced a transition to enjoyment and happiness during the activity, at least one participant did not overcome her nervousness. A minority of participants from both studies were explicitly aware of their reduced pain perception following interacting with the horses and mentioned this in their accounts.

Distraction involves a competition for attention, in this case between pain and a consciously directed focus on external stimuli, that is, a horse. While participating in the EAP, HI and EAL groups, participants were engaged in an attention-occupying active interaction between themselves and the two horses present with them. As mentioned previously, horses and ponies alike require a high level of attentiveness from their human counterpart due to their size and sensitivity to visual and auditory cues. In contrast although the Control Group participants did a similar task in the same environment, they did not have a horse/pony present. Rischer et al. (2020)^35^ conducted a study looking at high and low demand tasks in relation to painful stimuli. They found that those in the high load condition experienced significantly reduced perceived pain intensity when compared to the low load condition, and that the size of the distraction correlated with pain perception. Post-session pain rating were taken while still in the arena and the horses were still present. One way to explain the reduced pain perceived while with horses in the current study is therefore perhaps that the horses provide sufficient distraction.

The physiological changes that may have occurred during the experiment are suggested from previous research on AAT animal interaction.^36^ Furthermore increased levels of endorphins, as neurotransmitters, have been found to inhibit pain in the body, while also inhibiting positive feelings when at reduced levels.^37^ Hence, potential physiological changes may underlie some of the reduction in pain we see in our study. Future research could explore this. It is important to note that physiological changes, in particular endorphin release, also occur with any interesting or engaging activity,^38^ so this may not be just a horse/pony effect, but simply an outcome of participants enjoying themselves when interacting with the horses/ponies. Looking at the current qualitative data, a transition from nervous and anxious feelings to calm and relaxed feelings was observed in the EAP, HI and EAL groups.

Certainly horse-interaction provided participants with feelings of enjoyment and reward, with an overwhelmingly positive array of responses observed in those who participated in horse-interaction. These positive responses support research conducted by Braun et al. (2009)^39^ suggesting that AAT leads to the release of endorphins which promote a feeling of well-being. Positive emotion can lead to pain reduction^40^ giving a potential explanation that the significant reduction in pain perception observed in the horse-interaction groups ties in with the positive responses found in their qualitative descriptions. It remains to be seen whether the effect of such interactions is due to their novelty as well as whether the magnitude of pain perception reduction is or is not greater with horses in comparison to other inherently enjoyable experiences.

The results have shown that interaction with horses reduces pain perception, potentially through the processes of distraction, physiological changes, increased positive emotions and reduction in anxiety/increased relaxation. The Dynamic Model of Affect (DMA^11^), can aid the explanation of how these findings, on acute pain perception, can relate to the treatment of chronic pain. The DMA accounts for how positive states may influence adaptation to chronic pain. When applying the DMA to chronic pain populations, it was found that the ability to sustain positive states decreases vulnerability to negative pain and fosters adaptive recovery from pain. With horse-interaction possibly providing a positive outlet for emotion, it may be possible that horses could provide a positive state to those with chronic pain. It is also proposed that horse-interaction may provide a sufficient distraction to aid the relief from chronic pain to those who suffer from it. Indeed a participant in a horse condition commented that: ‘*as someone who suffers from endometriosis and PTSD, this study showed me that there is a chance for me to feel relief*’. Making horses available to patients has practical and monetary restrictions and it remains to be seen what the relative cost effectiveness of such interventions would be in comparison to other activities. Horses certainly command particular mindfulness when people are around them, partly because of their size,^41^ which may enhance a therapeutic effect.

Some of our participants expressed that their initial nervousness stemmed from the awareness of the large size of the horses. Interestingly we found similar effects when larger horses (Study 1) and smaller ponies (Study 2) took part. Frewin and Gardiner (2005)^42^ stated that the horses’ size can be intimidating for people, which engenders increased attentiveness and self-awareness around them. An advantage of this size differential is the opportunity to face one’s fears and insecurities, which translates to improved self-esteem resulting from managing horse-oriented tasks effectively.^43^ This is reflected in our findings, as many stated that successful interactions with the horses were a source of increased self-esteem and confidence. It is also well-documented in previous literature, being described as one of the most prominent benefits of EAP in many studies.^44^ For example, Wilson et al. (2017)^45^ interviewed eight EAP registered therapists offering EAP to adolescents diagnosed with depression and/or anxiety. When asked about the benefits they have observed in their patients, the most cited improvements were improved self-esteem and confidence. This was also the case in another sample of licensed therapists interviewed, with respondents claiming EAP facilitated increases in self-esteem and self-efficacy in depressed adolescents.^46^

There are a number of limitations of the current study. First, while participants were randomly allocated to the different groups, which were matched on age, we could not match groups also on other potentially important variables including previous experience with horses. All groups included some participants with little or no experience and some with extensive experience. However we did not collect data on this or quantify it. This would be an important focus for future research as previous experience might impact how participants respond to the EAL sessions, for example, by affecting novelty of the experience as well as feelings of anxiety. Second while we attempted to have adequate control groups in both studies, controlling for physical environment and activity levels, on reflection these could have been improved considerably by changing the task that participants were asked to complete. Future research should attempt to have a task that is inherently enjoyable (perhaps social) whether it is accomplished with or without the horses. This would hopefully reduce the negative feelings felt by some of the participants in the control group which may have created a confounding factor.

Future research can also investigate individual differences in responses to EAP. It has been well-documented that mental health conditions, such as depression, influence pain perception.^47^ It remains to be seen if the reductions in pain perception we found here would also occur across different mental health conditions. Furthermore, there is a need for research to investigate pain perception and horse-interaction with individuals who present with chronic pain conditions.

Lastly as discussed above, the majority of our participants reported feeling calmer, more relaxed and less nervous after the session, indicating a possible decrease in anxiety and stress. It would therefore be informative to include physiological measurements of anxiety and stress levels (e.g. heart rate, electrodermal activity) in future studies, in order to better understand the impact of human-horse interaction, and how this relates to changes in pain perception. Lastly, further research can examine whether more long-term participation in the EAP, HI and EAL groups would produce significant changes in pain tolerance over a sustained period of time.
